# Role of the microtubule-targeting drug vinflunine on cell-cell adhesions in bladder epithelial tumour cells

**DOI:** 10.1186/1471-2407-14-507

**Published:** 2014-07-10

**Authors:** Luis A Aparicio, Raquel Castosa, Mar Haz-Conde, Marta Rodríguez, Moisés Blanco, Manuel Valladares, Angélica Figueroa

**Affiliations:** 1 Translational Cancer Research Group, Instituto de Investigación Biomédica A Coruña (INIBIC), Complejo Hospitalario Universitario A Coruña (CHUAC), Sergas. As Xubias, 15006 A Coruña, España; 2 Servizo de Oncología Médica, Complejo Hospitalario Universitario A Coruña (CHUAC), Sergas, A Coruña, Spain

**Keywords:** Microtubule, Cell-cell contacts, E-cadherin, Vinflunine, Bladder cancer

## Abstract

**Background:**

Vinflunine (VFL) is a microtubule-targeting drug that suppresses microtubule dynamics, showing anti-metastatic properties both *in vitro* and in living cancer cells. An increasing body of evidence underlines the influence of the microtubules dynamics on the cadherin-dependent cell-cell adhesions. E-cadherin is a marker of epithelial-to-mesenchymal transition (EMT) and a tumour suppressor; its reduced levels in carcinoma are associated with poor prognosis. In this report, we investigate the role of VFL on cell-cell adhesions in bladder epithelial tumour cells.

**Methods:**

Human bladder epithelial tumour cell lines HT1376, 5637, SW780, T24 and UMUC3 were used to analyse cadherin-dependent cell-cell adhesions under VFL treatment. VFL effect on growth inhibition was measured by using a MTT colorimetric cell viability assay. Western blot, immunofluorescence and transmission electron microscopy analyses were performed to assess the roles of VFL effect on cell-cell adhesions, epithelial-to-mesenchymal markers and apoptosis. The role of the proteasome in controlling cell-cell adhesion was studied using the proteasome inhibitor MG132.

**Results:**

We show that VFL induces cell death in bladder cancer cells and activates epithelial differentiation of the remaining living cells, leading to an increase of E-cadherin-dependent cell-cell adhesion and a reduction of mesenchymal markers, such as N-cadherin or vimentin. Moreover, while E-cadherin is increased, the levels of Hakai, an E3 ubiquitin-ligase for E-cadherin, were significantly reduced in presence of VFL. In 5637, this reduction on Hakai expression was blocked by MG132 proteasome inhibitor, indicating that the proteasome pathway could be one of the molecular mechanisms involved in its degradation.

**Conclusions:**

Our findings underscore a critical function for VFL in cell-cell adhesions of epithelial bladder tumour cells, suggesting a novel molecular mechanism by which VFL may impact upon EMT and metastasis.

## Background

Bladder cancer is a common malignancy affecting the genitourinary system that represents the fifth most common cancer in the world. Transitional cell carcinoma (TCC) represents 95% of these tumours [1]. Most bladder cancers (70%-80%) present non-muscle invasive or superficial disease confined to the bladder mucosa (Ta) or lamina propria (T1), and the remaining (20%-30%) are muscle-invasive at the time of diagnosis (T2-T4) [2]. Although both bladder cancers originate from urothelium in the urinary bladder (the epithelium that lines the urinary tract), they have different clinical characteristics. Muscle invasive TCC of the bladder is associated with a high frequency of metastasis, resulting in poor prognosis for patients [3]. Therefore, an effective strategy for preventing the progression of bladder cancer is clearly needed.

Epithelial cells bind to each other, forming a strong adhesive cell layer with important barrier functions. Cell-cell contacts comprise different types of junctions, but adherens junctions are the major cell–cell junctions that mediate cell recognition, adhesion, morphogenesis, and tissue integrity. Adherens junctions are linked to the actin cytoskeleton, establishing molecular communication with other cell–cell junctions and cell–substratum adhesions, and are involved in the organization and movement of the cells within the epithelium and in the transmission of information to the interior of the cell. The most important mediators of cell-to-cell adhesion are the transmembrane proteins called cadherins. E- and N-cadherin were the first cadherins identified [4]. E-cadherin is the prototype and best-characterized member of adherens junctions in mammalian epithelial cells. It contains an extracellular domain that forms homophilic interactions in a calcium-dependent manner and is responsible for cell-to-cell adhesions, and a cytoplasmic domain linked to the actin cytoskeleton through its interaction with several catenins [5,6]. E-cadherin is regarded as a tumour suppressor and its loss is associated with poor prognosis in carcinoma.

E-cadherin is considered a hallmark of epithelial-to-mesenchymal transition (EMT). EMT is an early step during carcinoma metastasis characterized by the loss of epithelial morphology and the acquisition of mesenchymal and motile characteristics, resulting from the loss of apical-basal polarity, the loss of cell–cell contacts, and the reorganization of the actin cytoskeleton [7-9]. Numerous studies suggest that EMT is associated with cancer cell invasion, recurrence, progression and metastasis in various malignancies, including bladder cancer [10]. However, the EMT is a reversible transitional process, as the cells can return to their epithelial phenotype: a process that is known as mesenchymal-to-epithelial transition [11]. The loss of E-cadherin expression may also have a pivotal role in tumour progression characterized by increased mobility and invasiveness in bladder cancer [12-14]. Indeed, several studies on the prognostic role of E-cadherin in bladder cancer have shown that its aberrant expression is associated to tumour progression and poor prognosis [15]. A key change that occurs during EMT is the "cadherin switch", in which the normal expression of E-cadherin is replaced by the abnormal expression of N- or P-cadherin [16,17]. Another important marker frequently used in cells undergoing EMT during metastatic progression is vimentin. Vimentin is an intermediate filament protein that is also upregulated during EMT. Vimentin expression induces cell changes including mesenchymal cell shape, increased cell motility, and loss of adhesion in epithelial cells during EMT [18]. Other studies have also suggested that transcriptional and posttranscriptional regulators are involved in the control of EMT [19,20]. E-cadherin is also regulated at posttranslational level; Hakai was the first posttranslational regulator of E-cadherin stability [19,21]. Hakai is a RING finger-type E3 ubiquitin-ligase for the E-cadherin complex that mediates E-cadherin ubiquitination, endocytosis and degradation; in consequence, it disrupts cell-cell contacts. Moreover, many articles have described the emerging biological functions for Hakai protein pointing out its influence on tumour progression during EMT, proliferation, and oncogenesis [21-28].

The microtubule system, a major component of the cytoskeleton, was identified as a suitable target for cancer therapy, primarily based on their biological importance in coordinating chromosome segregation during mitosis. Microtubules are macromolecular filaments composed of tubulin. The clinical efficacy of the first-generation vinka alkaloid has prompted further research for novel analogues with improved clinical efficacy and safety. Such efforts have led to the development of vinflunine (VFL), a third-generation, semi-synthetic vinca alkaloid that, similar to other microtubule-targeting drugs, suppresses microtubule dynamics both *in vitro* and in living cancer cells [29,30]. In contrast to other vinca alkaloids, VFL shows superior antitumor activity and an excellent safety profile. VFL was approved by the European Medicines Agency (EMEA) as a second-line treatment for patients with urothelial carcinoma resistant to first-line platinum-containing chemotherapy [31,32]. VFL has shown anti-angiogenic, anti-vascular and anti-metastatic properties *in vitro* and *in vivo*[33]. Some potential underlying mechanisms of the anti-angiogenic property of microtubule targeting-agents have been reviewed [34,35]. Interestingly, in endothelial cells, it was shown that microtubule-targeting agents, including VFL, may produce their anti-migratory/anti-angiogenic effects through an increase in interphase microtubule dynamics. In endothelial cells, at low and non-cytotoxic concentrations, VFL inhibits cell motility [36].

Although cadherins are best understood to cooperate with the actin cytoskeleton, there is increasing evidence supporting a role of the microtubules in regulating cadherin biology. Indeed, the cross-talk between microtubule networks and cell-cell adhesion sites profoundly impact upon these structures and is essential for proper cell organization, polarization and motility [37-42]. In the current study, we wanted investigate the possible impact of the microtubule-targeting drug VFL on E-cadherin-based cell-cell adhesion, and to determine the possible influence on the EMT transition markers in epithelial bladder tumour cell lines. We describe that VFL induces cell death in bladder cancer cells and activates epithelial differentiation of the remaining living cells. It has an impact on cell-cell contact, leading to an increase E-cadherin dependent cell-cell adhesion, while reducing vimentin and N-cadherin mesenchymal markers. Moreover, the levels of the E3 ubiquitin-ligase Hakai were significantly reduced by VFL treatment in all cell lines tested. Moreover, this reduction in Hakai protein levels was recovered in presence of the proteasome inhibitor MG132 in 5637 cell line, suggesting that Hakai could be, at least, partially degraded in a proteasome-dependent manner. Our data suggest that VFL may be involved in a cross-talk between microtubule networks and cell-cell adhesion sites by its function as a microtubule-targeting drug, suggesting a novel molecular mechanism by which VFL may impact upon EMT and metastasis.

## Methods

### Cell culture and treatments

Human bladder epithelial tumour cell lines HT1376, 5637, UMUC3, SW780 and T24 were used. HT1376 cell line was obtained from American Type Culture Collections (Manassas, VA). UMUC3 and SW780 were generously donated by Dr. F. Garcia (Pharmamar S.A., Madrid) and 5637 and T24 by Dr. F. Real (Spanish National Cancer Research Centre from Madrid, Spain). HT1376 and UMUC3 cells were cultured in DMEM medium (Gibco, LifeTech), 5637 was cultured in RPMI medium (Gibco, LifeTech), SW780 was cultured in Leibovitz’s medium (Gibco, LifeTech) and T24 cell line was culture in McCoy’s 5A (Gibco, LifeTech); each media was supplemented with 100 U/ml penicillin, 100 μg/ml streptomycin, 1% L-glutamine and 10% foetal bovine serum. Cultures were maintained at 37ºC with 5% CO_2_ in a humidified incubator. HT1377 cells were grown in the indicated medium additionally supplemented with non-essential aminoacids (Gibco, LifeTech). A stock solution of vinflunine was prepared in distilled water. Cells were treated with VFL at the indicated final concentrations and for the times shown. MG132 was obtained from Sigma-Aldrich (St Louis, USA) and was added to the medium at final concentration of 20 μM for 2 hours.

### Antibodies and reagents

Antibodies were used that recognized the cytoplasmic portion of E-cadherin (Invitrogen, California, USA), Hakai (Hakai-2498, kindly provided by Dr. Yasuyuki Fujita [14]), N-cadherin (Abcam, Cambridge, UK), vimentin (Cell Signaling Technology, Massachussetts, USA), cyclin D1 (Santa Cruz Biotechnology, Texas, USA), and glyceraldehyde-3-phosphate dehydrogenase (GAPDH) (Invitrogen, California, USA). HRP-rabbit and mouse polyclonal antibodies were from GE Healthcare (Uppsala, Sweden) and Alexa Fluor 488 secondary antibody was from Invitrogen (UK). All antibodies were used at dilutions of 1:1000 for Western blot analysis, except for HRP-rabbit, mouse polyclonal antibodies, and anti-GAPDH antibodies that were used at 1:2000, 1:2000, and 1:10000 respectively. E-cadherin antibody (BD Bioscence, California, USA) was used for immunofluorescence at a dilution of 1:500 and Alexa Fluor 488 secondary antibody was used at a dilution of 1:100.

### Viability assay

For cytotoxicity assays, 1 × 10^4^ cells were plated per well into a 96-well plate and cultured for 24 h before treatment with VFL for 48 h. Serial dilutions of VFL dissolved in fresh medium were added to the cells in fresh medium. Growth inhibition of the epithelial tumour bladder cell lines was measured by using a MTT colorimetric cell viability assay kit (Sigma Aldrich, St Louis, MO) according to the manufacturer’s instructions. To measure absorbance at 570 nm, a Multiskan Plus Reader (Thermo Fisher, MA, USA) was used. The half-maximal inhibitory concentration (IC_50_) and the corresponding 95% confidence interval (95% CI) values were calculated from dose–response curves constructed using GraphPad Prism software. The data presented are the average of three independent experiments performed six times.

### Phase contrast microscopy

For phase-contrast images, 2 × 10^5^ cells were plated per well in a 6-well plate and treated with the indicated final concentrations of VFL (VFL) during 48 h. Cells were then fixed with 4% paraformaldehyde in phosphate-buffered saline (PBS) for 15 min. Phase-contrast images were acquired using a Nikon Eclipse-Ti microscope.

### Protein analysis

Protein was isolated using TriPure Reagent (Roche, Germany) according to the manufacturer’s instructions. Cell lysates (20 μg of proteins) were obtained by lysing cells in a buffer containing 1% Triton X-100 (20 mM Tris/HCl pH 7.5, 150 mM NaCl and 1% Triton X-100), a protease inhibitor cocktail (Sigma Aldrich, St Louise, USA), and 50 mM PMSF. Western blot analysis was performed as described previously [43].

### Transmission electron microscopy

For transmission electron microscopy, 5 × 10^4^ cells of 5637 bladder tumour cell line were plated into 0.4 μm-pore culture inserts (Corning 353095, USA) placed on a 24-well plate. After treatment with 5 μM of VFL during 48 hours, cells were fixed with 2.5% cold glutaraldehyde (Panreac, Spain) in 0.1 M sodium cacodylate buffer (Sigma-Aldrich, Germany) pH 7.4, for 16 h at 4°C. Inserts were postfixed in 1% osmium tetroxide in 0.1 M sodium cacodylate buffer, pH 7.4, for 1 h at room temperature, following by several washes with 0.1 M sodium cacodylate buffer and distilled water. Inserts were dehydrated in increasing concentrations of acetone and embedded in Spurr’s resin (Taab, Berkshire, UK). Ultrathin sections of 70– to 80-nm thickness were cut using an Ultracut-E ultramicrotome (Leica) and collected on formvar-coated copper mesh grids. Samples were examined with a JEOL JEM 1010 transmission electron microscope at 80 kV.

### Immunofluorescence and TUNEL assay

For immunofluorescence and TUNEL assay, 3 × 10^4^ cells were plated in chambers slides (Millipores, USA), fixed in 4% paraformaldehyde for 10 min, and then permeabilized in 0.5% Triton X-100-phosphate buffered saline (PBS) for 15 min. Cell death was measured by using Click-it TUNEL Alexa Fluor® 594 Imaging Assay (Invitrogen, UK) according to manufacturer’s instructions followed by blocking with BSA 3% in PBS for 1 h. Incubation with E-cadherin primary antibody for 1 h was followed by incubation in Alexa-Fluor 488-conjugated secondary antibody solution for 1 h. To visualize nuclei, it was used 4',6-Diamidino-2-Phenylindole, Dihydrochloride (DAPI, LifeTech, UK).Finally, the mounting media used was ProLong Gold antifade reagent (LifeTech, USA). Epifluorescence images were taken in Olympus microscope.

### RNA analysis

Total RNA was isolated using TriPure Reagent (Roche, Germany) according to manufacturer´s instruction. The immunoprecipitated RNA pellet was washed by following an alternative protocol described for small RNAs in RiboPure (Life Technologies, UK). The quality and quantity of the obtained RNA was determined by using Nanodrop ND-spectrophotometer (Thermo Fisher Scientific, MA, USA). For reverse transcription (RT), random hexamers and SuperScript first-strand Synthesis System for RT-PCR (Invitrogen, UK) were used. For mRNA analysis, real-time quantitative (q)PCR analysis was performed using gene-specific primers 5’-CGCAGACGAATTCCTATAAAGC-3’ and 5’- CCTTCTTCATCACCAGGTGG -3’ for human Hakai and 5’-TGACCTTGATTTATTTTGCATACC-3’ and 5’-CGAGCAAGACGTTCAGTCCT-3’ for HPRT. PCR was performed by using Light Cycler 480 SYBR Green I Master (Roche, Germany); amplification and quantification were carried out using a LightCycler 480 real-time lightcycler (Roche, Germany).

### Statistical analysis

Unless indicated, all experiments were analysed by using Students t-test to evaluate differences between treatments at the indicated significance levels.

## Results

### Vinflunine induces epithelial phenotype in bladder tumour cells

**Figure 1:**
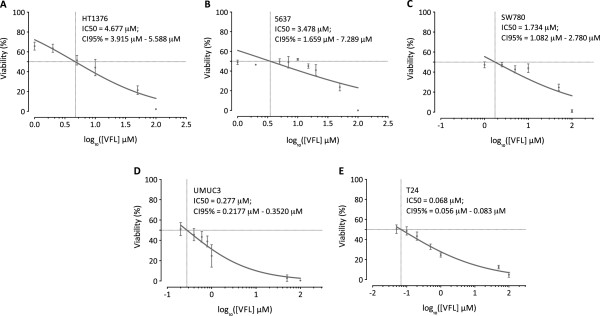
**Effect of VFL on cytotoxicity of bladder tumour cell lines.** The five indicated human tumour bladder cell lines (HT1376, 5637, SW780 in upper panel; UMUC3 and T24 in bottom panel) were treated with increasing concentrations of VFL (0–100 μM) for 48 h. Cell viability was determined by the MTT assay. **A**, HT1376. **B**, 5637. **C,** SW780. **D**, UMUC3 **E**, T24. Data are the means ± SEM of three independent experiments represented by logarithmic scale, and the IC50 value and CI95% for each cell line are indicated.

**Figure 2:**
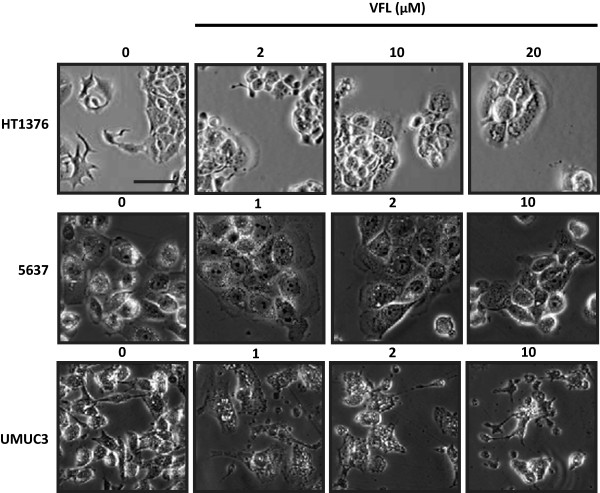
**Effect of VFL on the phenotype of bladder tumour cell lines.** Phase-contrast microscopy images of the indicated bladder cell lines taken 48 h after treatment with increasing concentrations of VFL compared to control conditions. Scale bar, 100 μm.

VFL, a microtubule-targeting drug, is used in monotherapy for treatment of advanced or metastatic urothelial cancers in adults. Given the rising evidence of crosstalk between microtubule networks and cell-cell adhesion sites, we sought to investigate the possible impact of VFL on cell-cell adhesions in bladder epithelial tumour cells. To this end, we first examined the effect of VFL on cell viability of HT1376, 5637, SW780, T24 and UMUC3 bladder epithelial tumour cells by using increasing concentrations of VFL (0–100 μM) treatment for 48 h. Figure 1 shows the dose-dependent inhibition of cell growth observed: IC_50_ = 4.677 μM for HT1376, IC_50_ = 3.478 μM for 5637 cells, IC_50_ = 1.734 μM for SW780, IC_50_ = 0.277 μM for UMUC3 cells and IC_50_ = 0.068 μM for T24, the latest cell lines showing the highest sensitivity to VFL. The cellular morphology following VFL treatment was analysed by phase-contrast microscopy in the indicated cell lines (Additional file 1 and Figure 2). As shown, HT1376 and 5637 showed drastic changes, with a morphology resembling that of epithelial cells, at VFL doses ranging between 1–20 μM, and tighter cell–cell contacts, as compared to control cells, which displayed a fibroblast-like morphology with decreased cell–cell contacts and increased numbers of membrane protrusions (Figure 2). This effect was also observed in SW780 tumor bladder cell line (data not shown). However, the fibroblastic morphology of UMUC3 and T24 cell lines was not affected by VFL treatment, showing an increased cell death, even in the presence of lower VFL concentrations (Figure 2 and data not shown). In conclusion, VFL affects the fibroblastic phenotype in HT1376, 5637 and SW780 bladder epithelial tumour cells, but not in UMUC3 and T24 cells.

### VFL effect on epithelial-to-mesenchymal transition markers

**Figure 3:**
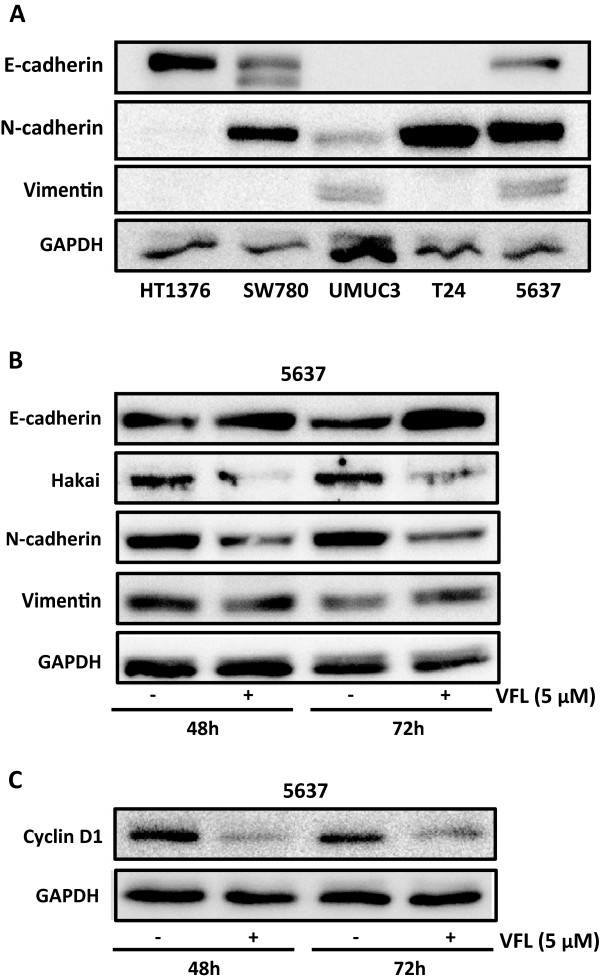
**Epithelial-to-mesenchymal markers. A**, the endogenous expression levels of the epithelial marker E-cadherin and mesenchymal markers N-cadherin and vimentin were assessed in bladder tumour cells (HT1376, SW780, UMUC3, T24 and 5637) by western blot analysis. **B**, the effect of 5 μM VFL treatments of 5637 bladder tumour cells for 48 and 72 h on the indicated protein markers was assessed by western blot analysis. **C**, Cyclin D1 expression levels were assessed by Western blot analysis after treatment of 5637 bladder tumour cells for 48 and 72 h with VFL. Western blot data are representative of three independent experiments and GAPDH antibody was used as loading control for normalization.

Given the different impact of VFL upon the presence of cell-cell contacts among the analysed bladder tumour cell lines, we set out to examine the endogenous levels of several EMT markers in the different bladder epithelial tumour cell lines. As shown in Figure 3A, important differences were found between the analysed cell lines. First, E-cadherin, a major epithelial marker that mediates cell-to-cell adhesions, was only detected in HT1376, 5637 and SW780 cells (Figure 3A), precisely the cell lines that were switched to a more epithelial-like phenotype and are more resistant to VFL treatment. Non E-cadherin expression was found in the cell lines that experienced the most cytotoxicity in response to VFL (Figure 3A), such as UMUC3 and T24. Interestingly, phenotypical changes under VFL treatments were not detected in UMUC3 and T24 cell lines (Figure 1). It was also analysed the expression level of N-cadherin and vimentin mesenchymal markers, which are frequently expressed in carcinoma cells that have undergone EMT. Together, these data suggest that E-cadherin expressing bladder tumour cells are more resistant to VFL and respond better to the VFL-triggered switch from mesenchymal-to-epithelial phenotype. Therefore, our results suggest that VFL can modulate cell death and epithelial cell differentiation.

**Figure 4:**
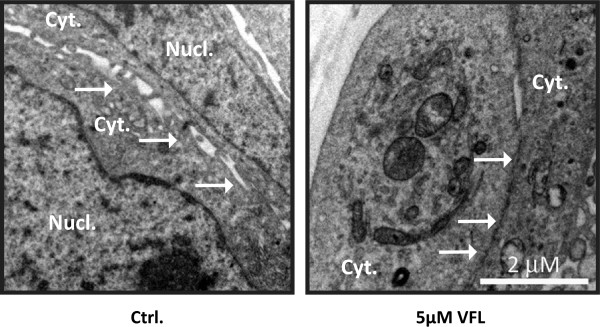
**Analysis of cell-cell contacts by transmission electron microscopy.** 5637 bladder cell lines were either untreated (left panel) or treated with 5 μM VFL 48 hours (right panel), whereupon cells were analysed by transmission electron microscopy. Nucl.: nucleus; Cyt: cytoplasm; Sites of close cell-cell contacts are shown (arrowheads),. Scale bar, 2 μm.

**Figure 5:**
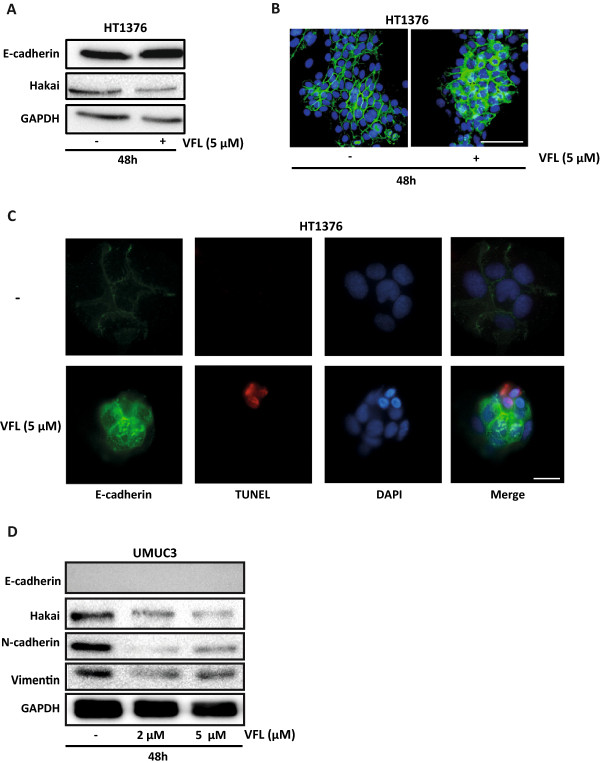
**Effect of VFL on epithelial differentiation and apoptosis. A**, Western blot analysis of E-cadherin and Hakai expression levels in HT1373 bladder tumour cells treated with 5 μM VFL for 48 h. **B**, immunofluorescence analysis of E-cadherin expression in HT1376 cells treated with 5 μM VFL for 48 hours. Scale bar, 200 μM. **C**, TUNEL staining for the analysis of apoptosis was performed following by immunofluorescence of E-cadherin in HT1376 cells treated with 5 μM VFL for 48 hours, as indicated in material and methods. Scale bar, 50 μM. **D**, Western blot analysis of the levels of E-cadherin, Hakai, N-cadherin and vimentin in UMUC3 bladder tumour cells treated with 5 μM VFL for 48 hours. Western blots are representative of three independent experiments and GAPDH was assessed as loading control.

VFL has an anti-metastatic property *in vitro* and *in vivo*; *in vitro* invasion assays showed an inhibitory effect of VFL treatment on invasion ability in a transitional cell carcinoma of the bladder. Moreover, in an orthotopic murine model of transitional cell carcinoma of the bladder, VFL showed potent high antitumor activity [44]. Since the initiation of metastasis requires invasion, which is enabled by EMT, we were interested in determining whether VFL might regulate the levels of EMT protein markers. A key change that occurs during EMT is the “cadherin switch”, in which the normal expression of E-cadherin is replaced by the abnormal expression of N-cadherin [16,17]. Downregulation of E-cadherin, responsible for the loss of cell-cell adhesions, and upregulation of mesenchymal-related proteins, such as vimentin or N-cadherin, define the EMT process [9]. As shown in Figure 3B, VFL treatment (5 μM) modestly increased protein expression of E-cadherin after 48 and 72 hours in 5637 bladder tumour cells; instead, the mesenchymal N-cadherin marker was reduced under the treatment. Moreover, the E3 ubiquitin-ligase Hakai for the E-cadherin complex was significantly reduced under these conditions, suggesting that the disappearance of Hakai protein could influence the recovery of E-cadherin expression. Hakai was also proposed to be involved in the regulation of both cell–cell contacts and cell proliferation. It was suggested that cyclin D1, a member of the cyclin protein family involved in the regulation of the cell cycle progression, was one of the substrate effector proteins through which Hakai might regulate cell proliferation [25]. Indeed, VFL treatment of 5637 cells caused a reduction in cyclin D1 protein levels compared to control conditions, while Hakai was also decreased (Figure 3C). In addition, transmission electron microscopy indicated that neighbouring VFL-treated E-cadherin expressing 5637 cells had very closely apposed cell-cell contacts compared to control cells (Figure 4). We extended this study in other bladder tumour epithelial cells. As shown in Figure 5A, in HT1376, VFL treatment modestly increases E-cadherin protein levels while Hakai is reduced; these cells do not express the mesenchymal markers vimentin or N-cadherin. By immunofluorescent staining, the VFL-elevated E-cadherin was detected at cell-cell contacts in epithelial cells (Figure 5B) while a reduction of E-cadherin protein at cell-cell was observed in cells undergoing apoptosis (Figure 5C). Finally, in UMUC3 cells, which do not express E-cadherin, it was shown that Hakai, vimentin, and N-cadherin levels were reduced after 48 h of vinflunine treatment (Figure 5D). Taken together, these data suggest that VFL causes cell death and epithelial cell differentiation in the E-cadherin-expressing cells.

### VFL promotes proteasome-mediated Hakai degradation

**Figure 6:**
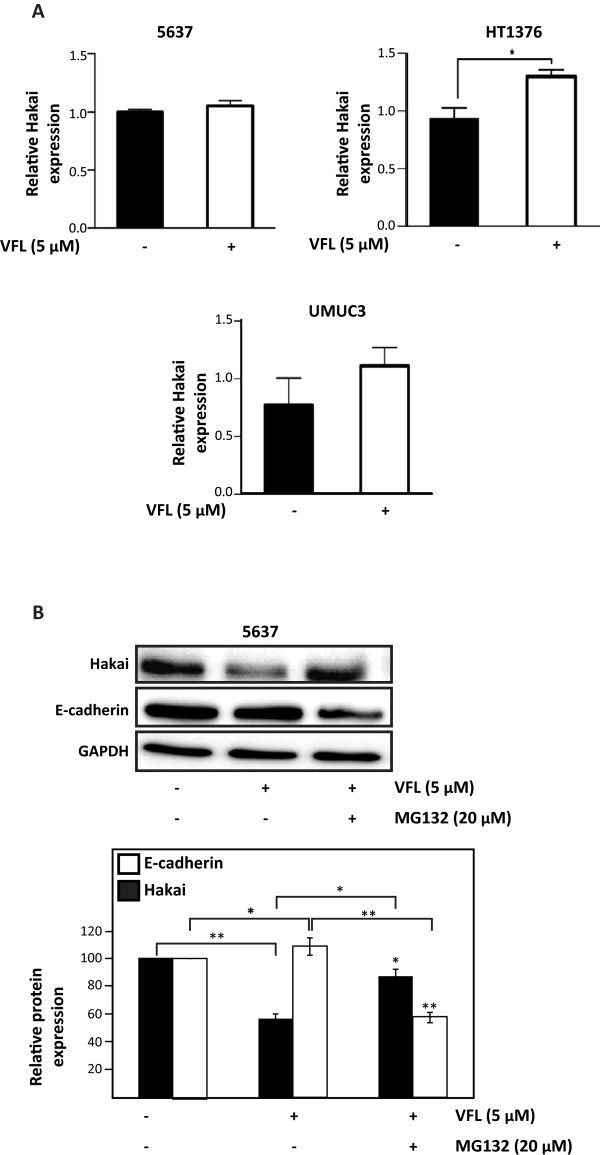
**VFL is involved in Hakai degradation via proteasome-mediated in 5637 cell line. A**, the levels of Hakai mRNA in 5637 (upper panel, left), HT1376 (upper panel, right), and UMUC3 (lower panel) cells following treatment with 5 μM VFL for 48 hours were determined by RT-qPCR analysis. HPRT mRNA levels were measured for normalization. The means ± SEM are represented from three independent experiments (*p < 0.05, n = 3). **B**, effect of proteasome inhibitor, MG132, on Hakai expression in 5637. Cells following treatment with 5 μM VFL for 48 hours were incubated for 2 h in the absence or presence of proteasome inhibitor (20 μM MG132) and cell lysates were prepared for Western blot analysis to detect Hakai, E-cadherin and normalization control GAPDH. Western blot data are representative of three independent experiments and quantification by densitometry was represented (lower panel, *p < 0.05, **p < 0.01).

Since VFL causes a reduction in Hakai protein levels, we examined whether VFL affects Hakai mRNA levels using reverse transcription (RT) followed by real-time, quantitative (q) PCR. In contrast with Hakai protein levels, Hakai mRNA levels were not downregulated by VFL treatment in 5637, HT1376 and UMUC3 (Figure 6A), suggesting that VFL lowers Hakai protein levels without decreasing Hakai mRNA abundance. Previous studies demonstrated that in all tissues, the majority of intracellular proteins are degraded by the ubiquitin proteasome pathway [45]. However, extracellular proteins and some cell surface proteins are taken up by endocytosis and degraded within lysosomes. Given that Hakai is an intracellular protein, we investigated whether the reduced Hakai levels in VFL-treated cells could be affected by the increased degradation via proteasome of Hakai protein. We analyzed the effect of the proteasome inhibitors MG132 in VFL-treated 5637 cells compared to control conditions. As shown in Figure 6B, treatment of 5637 bladder cancer cells with VFL consistently reduced Hakai protein levels; however, the addition of MG132 inhibited this VFL-mediated-Hakai down-regulation. As expected, given that Hakai protein levels are restored by MG132 treatment, E-cadherin is reduced under these conditions. In conclusion, in 5637 cell lines Hakai reduction can be recovered by using proteasome inhibitors, MG132, further supporting the notion that Hakai down-regulation induced by VFL can be at least partially controlled in a proteasome-dependent mechanism.

## Discussion

The transitional cell carcinomas of the bladder that invade muscle are associated with high frequency of metastasis, which is the major cause of death from cancer. Microtubules, a major component of the cytoskeleton, are one of the best established targets for cancer therapy. Indeed, the microtubule-targeting drug VFL, which suppresses microtubule dynamics *in vitro* and *in vivo*, is the recommended option treatment of metastatic transitional cell carcinoma of the urothelial tract which has progressed after treatment with platinum-containing chemotherapy. Given the increasing body of evidence supporting that microtubules regulate cadherin biology, and the well-established role of E-cadherin in the EMT during bladder cancer progression and metastasis, here we have studied the effect of VFL on E-cadherin cell-cell adhesions. Using bladder cancer cell lines, we have shown that VFL treatment induces cell death in bladder cancer cells and activates epithelial differentiation in the remaining cells, leading to increased E-cadherin-dependent cell-cell adhesions and to reduced levels of mesenchymal markers, such as N-cadherin or vimentin. Moreover, we have demonstrated that Hakai, a post-translational regulator of E-cadherin stability, was significantly reduced in a VFL-dependent manner in 5637 suggesting that the proteasome pathway is at least partially involved in its diminution; however, other post-translational mechanisms are waiting to be investigated. In conclusion, we have demonstrated a novel molecular mechanism of VFL to explain its anti-invasive effect.

In the last few years, differentiation therapy came out as a novel strategy for treating cancers. This approach is based on the concept that cancer cells arise from tissue stem cells and share the stemness and plasticity with normal stem cells. Differentiation therapy aims to induce cancer cells to differentiate by treatment with differentiation-inducing agents [46,47]. Most of the differentiation agents can inhibit proliferation and induce cells to differentiate and then undergo apoptosis [48,49]. It is well established that VFL blocks mitosis at the metaphase/anaphase transition, leading to apoptosis [50]. It is still necessary to elucidate how VFL could modulate epithelial cell differentiation and cell death; however, it appears to be an important therapeutic strategy for transitional cell carcinomas by its influence on these processes. Understanding the differentiation mechanisms and the fate of the treated cells may eventually lead us to gain insights into cancer therapy by differentiation.

Cadherins are key mediators of cell-to-cell adhesion in epithelial tissues. The roles of these proteins in bladder cancer-related EMT have been investigated extensively. Bryan and Tselepis summarized the patterns of P-cadherin and N-cadherin expression in the bladder during EMT [51]. In the normal urothelium, P-cadherin, but not N-cadherin, is expressed in the basal layer. However, during the EMT, P-cadherin expression in bladder cancer cells is upregulated along with N-cadherin expression; these events occur either independently or synchronously. E-cadherin expression in bladder cancer cells is lost after changes in P- and/or N-cadherin expression levels, as invasion and metastasis increase. This cadherin switching event is an important process that occurs late in the molecular pathogenesis of bladder cancer; although the precise timing and nature of these events remain unknown. On the other hand, the mesenchymal intermediate filament, vimentin, often increases its expression in carcinoma cells that have undergone an EMT. However, under VFL treatment, vimentin downregulation is observed in UMUC3 cell line but not in 5637. Vimentin levels are not always affected during the reversion of mesenchymal-to-epithelial transition. Indeed, partial EMT has been suggested to occur in some metastatic cancers, where distant metastasis can retain much of the epithelial differentiation of the original tissue of the cancer [52]. For instance, under silibinin treatment, a natural agent that reverses EMT, vimentin mesenchymal marker was not always influenced by this agent. Indeed, it was observed that vimentin was not always regulated by silibinin during MET and this modulation was dependent on the type of prostate epithelial tumor cells [53,54]. Various lines of evidence clearly indicate that the EMT is strongly associated with aggressive bladder cancer behavior, such as recurrence, progression, and metastasis, an observation that raises the possibility that the EMT may be a target for bladder cancer treatment. As mentioned above, E-cadherin is the first and most important regulator of the EMT. Moreover, in bladder cancer, loss of E-cadherin expression is a marker of poor response to the monoclonal antibody cetuximab, which blocks EGFR binding and represses bladder cancer cell proliferation [55]. Thus, E-cadherin expression levels could constitute an important predictive marker for the responsiveness to microtubule-targeting VFL therapy.

Several studies have demonstrated the influence of the microtubules on cadherin-dependent cell-cell adhesions. Kitase et. al. demonstrated that RhoA is implicated during neurodetermination, where it influences cell-cell contact and cadherin levels [56]. They used the P19 cell model of neuronal differentiation to show that RhoA affects cadherin protein level and cell-cell contacts during neuroinduction. RhoGTPases have an important role during neurite growth, axonal guidance, and synaptogenesis [57-59]. The cellular effects of RhoA are mediated by ROCK and seem to involve microtubules, pointing, for the first time, at the existence of a potential complex cross-talk between RhoA/ROCK, N-cadherin, and microtubules. The effect on cadherin level occurred in the timing that corresponds to the switch of E- to N-cadherin that trigger neurodifferentiation [60]. N-cadherin level appears to be critical for cell fate determination during morphogenesis. Advanced induction of Cdc42 had similar effects to RhoA inactivation, underscoring the importance of correct timing of RhoGTPases during neurodifferentiation [56].

Similar to VFL, another microtubule destabilizing agent, nocodazole, influenced cell-cell adhesions. The study reporting these findings examined the role of microtubules on the transcriptional regulation of cell adhesion proteins, providing evidence that the microtubule cytoskeleton critically affects EMT by regulating TGF-β/SMAD2 signaling during palatal fusion and prevents E-cadherin repression [61]. During palatal fusion, the midline epithelial seam (MES) degrades to achieve mesenchymal confluence. EMT is one of the mechanisms that function during MES degradation. TGF-β induces EMT in medial edge epithelium (MEE) by down-regulating the epithelial marker E-cadherin. Microtubule disassembly impaired palatal fusion leading to a multi-layered MES in the mid-region and inhibited palatal fusion accompanied by the development of a multi-layered MES in the mid-palatal region [62]. The authors further showed that treatment with nocodazole led to the accumulation of cell-cell adhesion proteins at intercellular junctions in medial edge epithelium [61]. Microtubule disruption by nocodazole triggered the aberrant accumulation of E-cadherin adhesion at intercellular junctions in MEE. Due to the aberrant expression of both negative (Snail and Zeb) and positive (c-MYC) E-cadherin transcriptional regulators when the TGF-β/SMAD2 signaling pathway was blocked, resulted in failure for EMT to progress. These data also support the important role of the microtubule cytoskeleton in mediating TGF-β/SMAD2 signals to control E-cadherin expression in MEE during palatal fusion [61].

Several lines of evidence support that the interaction of the microtubules with cadherin affects cadherin biology [63]. First, the junctional integrity of cadherin is perturbed by drugs such as nocodazole and VFL, which disrupt microtubules. Second, specific targeting of microtubule-binding proteins found at junctions also impairs broad aspects of cadherin biology. For example, Nezha-bound microtubules at their minus ends and tethered them to the zonula adherens. In cultured mammalian epithelial cells, depletion of either Nezha or PLEKHA7, which is responsible for recruiting Nezha to p120-ctn, disrupted the ability of cells to concentrate E-cadherin to the apical junction of the zonula adherens. The functional impact of these junctional microtubule-binding proteins further supports the idea that microtubules that interact with cadherin adhesions are responsible for regulating junctional integrity. Still, how microtubules influence these diverse aspects of cadherin biology is complex and poorly understood. Microtubules are commonly implicated in directing intracellular traffic of membrane-bound vesicles or molecular complexes; accordingly, it was postulated to influence cadherins through their intracellular traffic. Indeed, a number of microtubule-based motors have been identified that support intracellular transport of cadherins [64].

The EMT is regarded as a key process that allows cancer cells to migrate to adjacent organs or metastasize to distant sites. In bladder cancer, EMT is closely associated with grave clinical characteristics, such as recurrence, progression, metastasis, and reduced survival. However, metastases of the most common human cancers (well- to moderately-differentiated carcinomas) often show a re-differentiation in the sense of a mesenchymal-epithelial transition (MET). Therefore, strategies to induce reversal EMT (MET), such as VFL, might be able to suppress cancer cell migration and metastasis. However, this issue is controversial, as several lines of evidence support that transient dedifferentiation (EMT) and re-differentiation (MET) processes are a driving force in metastasis [65]. Although many clinical reports fostered the concept of transient EMT-MET switches in metastasis, only little experimental evidence is available [66]. Chaffer et al. support the role of an EMT in dissemination and the need of a MET for efficient metastasis in bladder cancer metastasis [66]. Two reports support the need of re-differentiation (MET) for the colonization and metastasis of differentiated carcinomas and implicate EMT-associated growth arrest in these events [67-69]. Therefore, MET has a key clinical impact for future therapeutic strategies against metastasis. On one hand, EMT-targeting therapy may be useful as a personalized medicine approach that complements conventional bladder cancer treatments, while on the other hand, the induction of differentiation and targeting EMT alone might be counterproductive by activating the proliferation of disseminated cells. It is possible that therapeutic approaches directed against EMT can benefit cancer patients diagnosed at early stages of the disease to prevent invasion and dissemination, while anti-MET drugs could be potentially benefit patients with established metastasis. Still, a better understanding of the molecular mechanisms of the EMT in bladder cancer is crucial to the development of new therapeutic modalities for this cancer [69,70].

## Conclusions

Our data suggest that VFL, a microtubule-targeting drug, may be involved in a cross-talk between microtubule networks and cell-cell adhesions. We suggest a novel molecular mechanism by which VFL may impact upon EMT and metastasis. VFL impacts on E-cadherin-based cell-cell adhesion and influence on the EMT markers in epithelial bladder tumour cell lines. We have shown that VFL induces cell death in bladder cancer cells and activates epithelial differentiation of the remaining living cells. VFL increases E-cadherin dependent cell-cell adhesion, and reduces vimentin and N-cadherin mesenchymal markers. Moreover, the levels of the E3 ubiquitin-ligase Hakai were reduced by VFL in 5637 suggesting that proteasome-dependent mechanism could be one of the molecular mechanisms implicated in this reduction. Our findings suggest the existence of a new mechanism of VFL-microtubule targeting drug to cell-cell contacts with potential functional implications in the maintenance of epithelial cell phenotype.

## Abbreviations

EMT: Epithelial-to-mesenchymal transition; MEE: Medial edge epithelium; MET: Mesenchymal-to-epithelial transition; TCC: Transitional cell carcinoma; VFL: Vinflunine.

## Competing interests

The authors declare that they have no competing interests.

## Authors’ contributions

RC, MHC and MR contributed to experimental research. AF contributed to the design, the analysis of the results and writing the manuscript. All the authors have given the discussion, critical reading of the manuscript and final approval of the version to be published.

## Pre-publication history

The pre-publication history for this paper can be accessed here:

http://www.biomedcentral.com/1471-2407/14/507/prepub

## Supplementary Material

Additional file 1 **Figure for reviewer 3.** Effect of VFL on the phenotype of 5637 and HT1376 bladder tumour cell lines. Phase-contrast microscopy images, showing larger fields from images of Figure 2, of the indicated bladder cell lines taken 48 h after treatment with the indicated concentrations of VFL compared to control conditions. Click here for file

## References

[B1] Siegel R Naishadham D Jemal A Cancer statistics, 2012 CA Cancer J Clin 2012 62 1 10 29 22237781 10.3322/caac.20138

[B2] Raghavan D Shipley WU Garnick MB Russell PJ Richie JP Biology and management of bladder cancer N Engl J Med 1990 322 16 1129 1138 2181313 10.1056/NEJM199004193221607

[B3] Stenzl A Cowan NC De Santis M Jakse G Kuczyk MA Merseburger AS Ribal MJ Sherif A Witjes JA The updated EAU guidelines on muscle-invasive and metastatic bladder cancer Eur Urol 2009 55 4 815 825 19157687 10.1016/j.eururo.2009.01.002

[B4] Nollet F Kools P van Roy F Phylogenetic analysis of the cadherin superfamily allows identification of six major subfamilies besides several solitary members J Mol Biol 2000 299 3 551 572 10835267 10.1006/jmbi.2000.3777

[B5] Perez-Moreno M Jamora C Fuchs E Sticky business: orchestrating cellular signals at adherens junctions Cell 2003 112 4 535 548 12600316 10.1016/s0092-8674(03)00108-9

[B6] Yonemura S Cadherin-actin interactions at adherens junctions Curr Opin Cell Biol 2011 23 5 515 522 21807490 10.1016/j.ceb.2011.07.001

[B7] Christofori G New signals from the invasive front Nature 2006 441 7092 444 450 16724056 10.1038/nature04872

[B8] Yang J Weinberg RA Epithelial-mesenchymal transition: at the crossroads of development and tumor metastasis Dev Cell 2008 14 6 818 829 18539112 10.1016/j.devcel.2008.05.009

[B9] Thiery JP Acloque H Huang RY Nieto MA Epithelial-mesenchymal transitions in development and disease Cell 2009 139 5 871 890 19945376 10.1016/j.cell.2009.11.007

[B10] Yun SJ Kim WJ Role of the Epithelial-Mesenchymal Transition in Bladder Cancer: From Prognosis to Therapeutic Target Korean J Urol 2013 54 10 645 650 24175036 10.4111/kju.2013.54.10.645 PMC3806986

[B11] Chen J Han Q Pei D EMT and MET as paradigms for cell fate switching J Mol Cell Biol 2012 4 2 66 69 22140271 10.1093/jmcb/mjr045

[B12] Birchmeier W Behrens J Cadherin expression in carcinomas: role in the formation of cell junctions and the prevention of invasiveness Biochim Biophys Acta 1994 1198 1 11 26 8199193 10.1016/0304-419x(94)90003-5

[B13] Behrens J Vakaet L Friis R Winterhager E Van Roy F Mareel MM Birchmeier W Loss of epithelial differentiation and gain of invasiveness correlates with tyrosine phosphorylation of the E-cadherin/beta-catenin complex in cells transformed with a temperature-sensitive v-SRC gene J Cell Biol 1993 120 3 757 766 8425900 10.1083/jcb.120.3.757 PMC2119534

[B14] Perl AK Wilgenbus P Dahl U Semb H Christofori G A causal role for E-cadherin in the transition from adenoma to carcinoma Nature 1998 392 6672 190 193 9515965 10.1038/32433

[B15] Shimazui T Schalken JA Giroldi LA Jansen CF Akaza H Koiso K Debruyne FM Bringuier PP Prognostic value of cadherin-associated molecules (alpha-, beta-, and gamma-catenins and p120cas) in bladder tumors Cancer Res 1996 56 18 4154 4158 8797585

[B16] De Wever O Pauwels P De Craene B Sabbah M Emami S Redeuilh G Gespach C Bracke M Berx G Molecular and pathological signatures of epithelial-mesenchymal transitions at the cancer invasion front Histochem Cell Biol 2008 130 3 481 494 18648847 10.1007/s00418-008-0464-1 PMC2522326

[B17] Yilmaz M Christofori G EMT, the cytoskeleton, and cancer cell invasion Cancer Metastasis Rev 2009 28 1–2 15 33 19169796 10.1007/s10555-008-9169-0

[B18] Mendez MG Kojima S Goldman RD Vimentin induces changes in cell shape, motility, and adhesion during the epithelial to mesenchymal transition FASEB J 2010 24 6 1838 1851 20097873 10.1096/fj.09-151639 PMC2874471

[B19] Aparicio LA Valladares M Blanco M Alonso G Figueroa A Biological influence of Hakai in cancer: a 10-year review Cancer Metastasis Rev 2012 31 1-2 375 386 22349934 10.1007/s10555-012-9348-x PMC3350634

[B20] Batlle E Sancho E Francí C Domínguez D Monfar M Baulida J García De Herreros A The transcription factor snail is a repressor of E-cadherin gene expression in epithelial tumour cells Nat Cell Biol 2000 2 2 84 89 10655587 10.1038/35000034

[B21] Fujita Y Krause G Scheffner M Zechner D Leddy H Behrens J Sommer T Birchmeier W Hakai, a c-Cbl-like protein, ubiquitinates and induces endocytosis of the E-cadherin complex Nat Cell Biol 2002 4 3 222 231 11836526 10.1038/ncb758

[B22] Swaminathan G Cartwright CA Rack1 promotes epithelial cell-cell adhesion by regulating E-cadherin endocytosis Oncogene 2011 31 3 376 389 21685945 10.1038/onc.2011.242

[B23] Janda E Nevolo M Lehmann K Downward J Beug H Grieco M Raf plus TGFbeta-dependent EMT is initiated by endocytosis and lysosomal degradation of E-cadherin Oncogene 2006 25 54 7117 7130 16751808 10.1038/sj.onc.1209701

[B24] Zhou WJ Geng ZH Chi S Zhang W Niu XF Lan SJ Ma L Yang X Wang LJ Ding YQ Geng JG Slit-Robo signaling induces malignant transformation through Hakai-mediated E-cadherin degradation during colorectal epithelial cell carcinogenesis Cell Res 2011 21 4 609 626 21283129 10.1038/cr.2011.17 PMC3203654

[B25] Figueroa A Kotani H Toda Y Mazan-Mamczarz K Mueller E Otto A Disch L Norman M Ramdasi R Keshtgar M Gorospe M Fujita Y Novel roles of hakai in cell proliferation and oncogenesis Mol Biol Cell 2009 20 15 3533 3542 19535458 10.1091/mbc.E08-08-0845 PMC2719571

[B26] Figueroa A Fujita Y Gorospe M Hacking RNA: Hakai promotes tumorigenesis by enhancing the RNA-binding function of PSF Cell Cycle 2009 8 22 3648 3651 19855157 10.4161/cc.8.22.9909 PMC2808762

[B27] Rodríguez-Rigueiro T Valladares-Ayerbes M Haz-Conde M Aparicio LA Figueroa A Hakai reduces cell-substratum adhesion and increases epithelial cell invasion BMC Cancer 2011 11 474 22051109 10.1186/1471-2407-11-474 PMC3229560

[B28] Abella V Valladares M Rodriguez T Haz M Blanco M Tarrío N Iglesias P Aparicio LA Figueroa A miR-203 Regulates Cell Proliferation through Its Influence on Hakai Expression PLoS One 2012 7 12 e52568 23285092 10.1371/journal.pone.0052568 PMC3527564

[B29] Jordan MA Horwitz SB Lobert S Correia JJ Exploring the mechanisms of action of the novel microtubule inhibitor vinflunine Semin Oncol 2008 35 3 Suppl 3 S6 S12 18538179 10.1053/j.seminoncol.2008.01.009

[B30] Jordan MA Wilson L Microtubules as a target for anticancer drugs Nat Rev Cancer 2004 4 4 253 265 15057285 10.1038/nrc1317

[B31] Gerullis H Ecke T Eimer C Wishahi M Otto T Vinflunine as second-line treatment in platin-resistant metastatic urothelial carcinoma: a review Anticancer Drugs 2011 22 1 9 17 20948429 10.1097/CAD.0b013e3283404db0

[B32] Gerullis H Vinflunine: a fluorinated vinca alkaloid for bladder cancer therapy Drugs Today (Barc) 2011 47 1 17 25 21373647 10.1358/dot.2011.47.1.1576693

[B33] Aparicio LM Pulido EG Gallego GA Vinflunine: a new vision that may translate into antiangiogenic and antimetastatic activity Anticancer Drugs 2012 23 1 1 11 22027536 10.1097/CAD.0b013e32834d237b

[B34] Pasquier E Honoré S Braguer D Microtubule-targeting agents in angiogenesis: where do we stand? Drug Resist Updat 2006 9 1–2 74 86 16714139 10.1016/j.drup.2006.04.003

[B35] Schwartz EL Antivascular actions of microtubule-binding drugs Clin Cancer Res 2009 15 8 2594 2601 19351751 10.1158/1078-0432.CCR-08-2710 PMC2745203

[B36] Honoré S Pagano A Gauthier G Bourgarel-Rey V Verdier-Pinard P Civiletti K Kruczynski A Braguer D Antiangiogenic vinflunine affects EB1 localization and microtubule targeting to adhesion sites Mol Cancer Ther 2008 7 7 2080 2089 18645018 10.1158/1535-7163.MCT-08-0156

[B37] Akhmanova A Yap AS Organizing junctions at the cell-cell interface Cell 2008 135 5 791 793 19041742 10.1016/j.cell.2008.11.002

[B38] Akhmanova A Stehbens SJ Yap AS Touch, grasp, deliver and control: functional cross-talk between microtubules and cell adhesions Traffic 2009 10 3 268 274 19175539 10.1111/j.1600-0854.2008.00869.x

[B39] Stehbens SJ Paterson AD Crampton MS Shewan AM Ferguson C Akhmanova A Parton RG Yap AS Dynamic microtubules regulate the local concentration of E-cadherin at cell-cell contacts J Cell Sci 2006 119 Pt 9 1801 1811 16608875 10.1242/jcs.02903

[B40] Stehbens SJ Akhmanova A Yap AS Microtubules and cadherins: a neglected partnership Front Biosci (Landmark Ed) 2009 14 3159 3167 19273264 10.2741/3442

[B41] Han SP Yap AS The cytoskeleton and classical cadherin adhesions Subcell Biochem 2012 60 111 135 22674070 10.1007/978-94-007-4186-7_6

[B42] Shtutman M Chausovsky A Prager-Khoutorsky M Schiefermeier N Boguslavsky S Kam Z Fuchs E Geiger B Borisy GG Bershadsky AD Signaling function of alpha-catenin in microtubule regulation Cell Cycle 2008 7 15 2377 2383 18677116 10.4161/cc.6362 PMC2668206

[B43] Rodríguez Rigueiro T Valladares Ayerbes M Haz Conde M Blanco M Aparicio G Fernández Puente P Blanco FJ Lorenzo MJ Aparicio LA Figueroa A A novel procedure for protein extraction from formalin-fixed paraffin-embedded tissues Proteomics 2011 11 12 2555 2559 21591256 10.1002/pmic.201000809

[B44] Bonfil RD Russo DM Binda MM Delgado FM Vincenti M Higher antitumor activity of vinflunine than vinorelbine against an orthotopic murine model of transitional cell carcinoma of the bladder Urol Oncol 2002 7 4 159 166 12474532 10.1016/s1078-1439(02)00184-9

[B45] Rock KL Gramm C Rothstein L Clark K Stein R Dick L Hwang D Goldberg AL Inhibitors of the proteasome block the degradation of most cell proteins and the generation of peptides presented on MHC class I molecules Cell 1994 78 5 761 771 8087844 10.1016/s0092-8674(94)90462-6

[B46] Zhang Z Zhou Y Qian H Shao G Lu X Chen Q Sun X Chen D Yin R Zhu H Shao Q Xu W Stemness and inducing differentiation of small cell lung cancer NCI-H446 cells Cell Death Dis 2013 4 e633 23681228 10.1038/cddis.2013.152 PMC3674360

[B47] Guichet PO Bieche I Teigell M Serguera C Rothhut B Rigau V Scamps F Ripoll C Vacher S Taviaux S Chevassus H Duffau H Mallet J Susini A Joubert D Bauchet L Hugnot JP Cell death and neuronal differentiation of glioblastoma stem-like cells induced by neurogenic transcription factors Glia 2013 61 2 225 239 23047160 10.1002/glia.22429

[B48] Campos B Wan F Farhadi M Ernst A Zeppernick F Tagscherer KE Ahmadi R Lohr J Dictus C Gdynia G Combs SE Goidts V Helmke BM Eckstein V Roth W Beckhove P Lichter P Unterberg A Radlwimmer B Herold-Mende C Differentiation therapy exerts antitumor effects on stem-like glioma cells Clin Cancer Res 2010 16 10 2715 2728 20442299 10.1158/1078-0432.CCR-09-1800

[B49] Seigel GM Differentiation potential of human retinoblastoma cells Curr Pharm Biotechnol 2011 12 2 213 216 21044005 10.2174/138920111794295846

[B50] Okouneva T Hill BT Wilson L Jordan MA The effects of vinflunine, vinorelbine, and vinblastine on centromere dynamics Mol Cancer Ther 2003 2 5 427 436 12748304

[B51] Bryan RT Tselepis C Cadherin switching and bladder cancer J Urol 2010 184 2 423 431 20620393 10.1016/j.juro.2010.04.016

[B52] Debnath J Brugge JS Modelling glandular epithelial cancers in three-dimensional cultures Nat Rev Cancer 2005 5 9 675 688 16148884 10.1038/nrc1695

[B53] Deep G Gangar S Agarwal C Agarwal R Role of E-cadherin in anti-migratory and anti-invasive efficacy of silibinin in prostate cancer cells Cancer Prev Res (Phila) 2011 4 8 1222 1232 21546539 10.1158/1940-6207.CAPR-10-0370 PMC3151351

[B54] Wu K Zeng J Li L Fan J Zhang D Xue Y Zhu G Yang L Wang X He D Silibinin reverses epithelial-to-mesenchymal transition in metastatic prostate cancer cells by targeting transcription factors Oncol Rep 2010 23 6 1545 1552 20428808

[B55] Black PC Brown GA Inamoto T Shrader M Arora A Siefker-Radtke AO Adam L Theodorescu D Wu X Munsell MF Bar-Eli M McConkey DJ Dinney CP Sensitivity to epidermal growth factor receptor inhibitor requires E-cadherin expression in urothelial carcinoma cells Clin Cancer Res 2008 14 5 1478 1486 18316572 10.1158/1078-0432.CCR-07-1593

[B56] Laplante I Béliveau R Paquin J RhoA/ROCK and Cdc42 regulate cell-cell contact and N-cadherin protein level during neurodetermination of P19 embryonal stem cells J Neurobiol 2004 60 3 289 307 15281068 10.1002/neu.20036

[B57] Lehmann M Fournier A Selles-Navarro I Dergham P Sebok A Leclerc N Tigyi G McKerracher L Inactivation of Rho signaling pathway promotes CNS axon regeneration J Neurosci 1999 19 17 7537 7547 10460260 10.1523/JNEUROSCI.19-17-07537.1999 PMC6782492

[B58] Luo L Rho GTPases in neuronal morphogenesis Nat Rev Neurosci 2000 1 3 173 180 11257905 10.1038/35044547

[B59] Yuan XB Jin M Xu X Song YQ Wu CP Poo MM Duan S Signalling and crosstalk of Rho GTPases in mediating axon guidance Nat Cell Biol 2003 5 1 38 45 12510192 10.1038/ncb895

[B60] Gao X Bian W Yang J Tang K Kitani H Atsumi T Jing N A role of N-cadherin in neuronal differentiation of embryonic carcinoma P19 cells Biochem Biophys Res Commun 2001 284 5 1098 1103 11414696 10.1006/bbrc.2001.5089

[B61] Kitase Y Shuler CF Microtubule disassembly prevents palatal fusion and alters regulation of the E-cadherin/catenin complex Int J Dev Biol 2013 57 1 55 60 23585353 10.1387/ijdb.120117yk

[B62] Kitase Y Shuler CF Multi-layered hypertrophied MEE formation by microtubule disruption via GEF-H1/RhoA/ROCK signaling pathway Dev Dyn 2012 241 7 1169 1182 22565548 10.1002/dvdy.23800

[B63] Brieher WM Yap AS Cadherin junctions and their cytoskeleton(s) Curr Opin Cell Biol 2013 25 1 39 46 23127608 10.1016/j.ceb.2012.10.010

[B64] Meng W Mushika Y Ichii T Takeichi M Anchorage of microtubule minus ends to adherens junctions regulates epithelial cell-cell contacts Cell 2008 135 5 948 959 19041755 10.1016/j.cell.2008.09.040

[B65] Brabletz T Jung A Reu S Porzner M Hlubek F Kunz-Schughart LA Knuechel R Kirchner T Variable beta-catenin expression in colorectal cancers indicates tumor progression driven by the tumor environment Proc Natl Acad Sci U S A 2001 98 18 10356 10361 11526241 10.1073/pnas.171610498 PMC56965

[B66] Chaffer CL Brennan JP Slavin JL Blick T Thompson EW Williams ED Mesenchymal-to-epithelial transition facilitates bladder cancer metastasis: role of fibroblast growth factor receptor-2 Cancer Res 2006 66 23 11271 11278 17145872 10.1158/0008-5472.CAN-06-2044

[B67] Ocaña OH Córcoles R Fabra A Moreno-Bueno G Acloque H Vega S Barrallo-Gimeno A Cano A Nieto MA Metastatic colonization requires the repression of the epithelial-mesenchymal transition inducer Prrx1 Cancer Cell 2012 22 6 709 724 23201163 10.1016/j.ccr.2012.10.012

[B68] Tsai JH Donaher JL Murphy DA Chau S Yang J Spatiotemporal regulation of epithelial-mesenchymal transition is essential for squamous cell carcinoma metastasis Cancer Cell 2012 22 6 725 736 23201165 10.1016/j.ccr.2012.09.022 PMC3522773

[B69] Brabletz T EMT and MET in metastasis: where are the cancer stem cells? Cancer Cell 2012 22 6 699 701 23238008 10.1016/j.ccr.2012.11.009

[B70] Nieto MA Cano A The epithelial-mesenchymal transition under control: global programs to regulate epithelial plasticity Semin Cancer Biol 2012 22 5–6 361 368 22613485 10.1016/j.semcancer.2012.05.003

