# Supplementary material for: Role of the microtubule-targeting drug vinflunine on cell-cell adhesions in bladder epithelial tumour cells
Source: BMC Cancer. 2014 Jul 10;14:507. doi: 10.1186/1471-2407-14-507 (PMC4107965; doi:10.1186/1471-2407-14-507)
Supplement: Additional file 1 — Figure for reviewer 3. Effect of VFL on the phenotype of 5637 and HT1376 bladder tumour cell lines. Phase-contrast microscopy images, showing larger fields from images of Figure 2, of the indicated bladder cell lines taken 48 h after treatment with the indicated concentrations of VFL compared to control conditions. [file 1471-2407-14-507-S1.pdf]

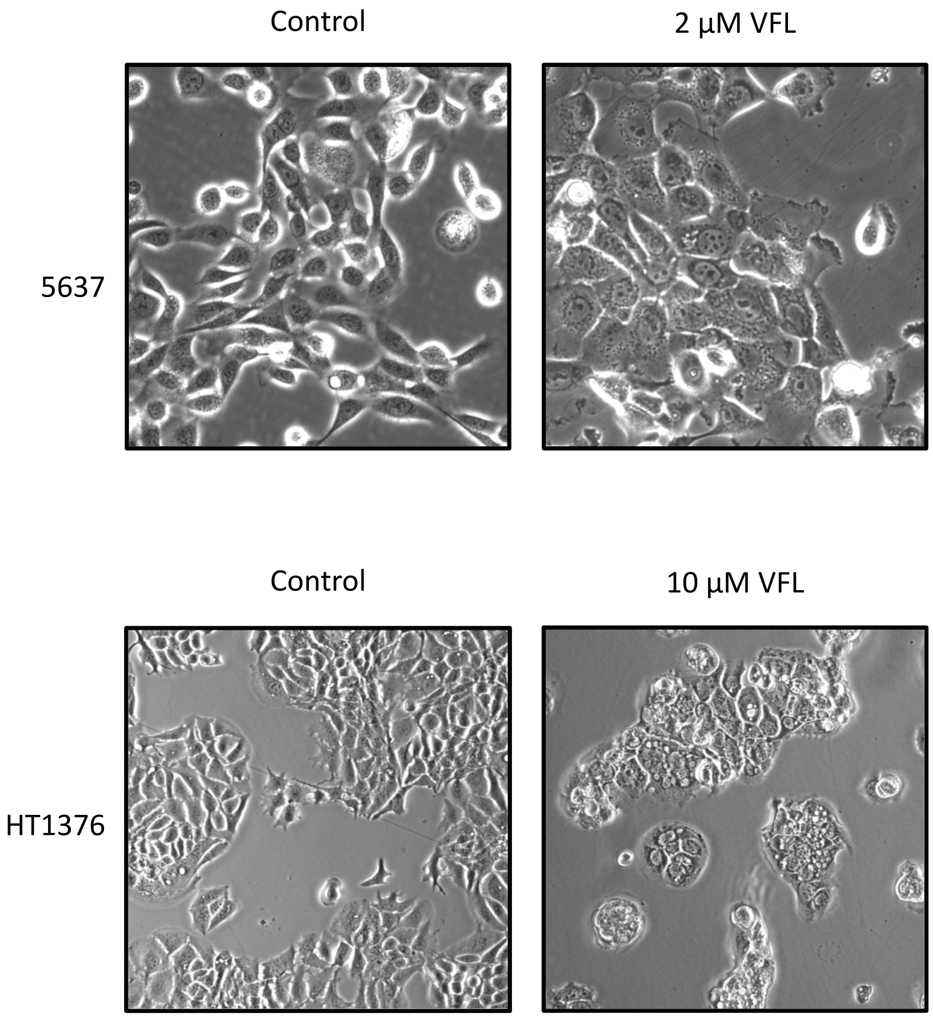

Figure for reviewer 3.
